# Passive Mobile Self-tracking of Mental Health by Veterans With Serious Mental Illness: Protocol for a User-Centered Design and Prospective Cohort Study

**DOI:** 10.2196/39010

**Published:** 2022-08-05

**Authors:** Alexander S Young, Abigail Choi, Shay Cannedy, Lauren Hoffmann, Lionel Levine, Li-Jung Liang, Melissa Medich, Rebecca Oberman, Tanya T Olmos-Ochoa

**Affiliations:** 1 Semel Institute for Neuroscience & Human Behavior Department of Psychiatry and Biobehavioral Sciences University of California, Los Angeles Los Angeles, CA United States; 2 Veterans Integrated Service Network-22 Mental Illness Research, Education and Clinical Center Greater Los Angeles Veterans Healthcare System Department of Veterans Affairs Los Angeles, CA United States; 3 Henry Samueli School of Engineering and Applied Science University of California, Los Angeles Los Angeles, CA United States; 4 Department of Medicine David Geffen School of Medicine University of California, Los Angeles Los Angeles, CA United States; 5 Center for the Study of Healthcare Innovation, Implementation & Policy Greater Los Angeles Veterans Healthcare Center Department of Veterans Affairs Los Angeles, CA United States

**Keywords:** serious mental illness, mobile health, mental health, passive sensing, health informatics, behavior, sensor, self-tracking, predict, assessment

## Abstract

**Background:**

Serious mental illnesses (SMI) are common, disabling, and challenging to treat, requiring years of monitoring and treatment adjustments. Stress or reduced medication adherence can lead to rapid worsening of symptoms and behaviors. Illness exacerbations and relapses generally occur with little or no clinician awareness in real time, leaving limited opportunity to modify treatments. Previous research suggests that passive mobile sensing may be beneficial for individuals with SMI by helping them monitor mental health status and behaviors, and quickly detect worsening mental health for prompt assessment and intervention. However, there is too little research on its feasibility and acceptability and the extent to which passive data can predict changes in behaviors or symptoms.

**Objective:**

The aim of this research is to study the feasibility, acceptability, and safety of passive mobile sensing for tracking behaviors and symptoms of patients in treatment for SMI, as well as developing analytics that use passive data to predict changes in behaviors and symptoms.

**Methods:**

A mobile app monitors and transmits passive mobile sensor and phone utilization data, which is used to track activity, sociability, and sleep in patients with SMI. The study consists of a user-centered design phase and a mobile sensing phase. In the design phase, focus groups, interviews, and usability testing inform further app development. In the mobile sensing phase, passive mobile sensing occurs with participants engaging in weekly assessments for 9 months. Three- and nine-month interviews study the perceptions of passive mobile sensing and ease of app use. Clinician interviews before and after the mobile sensing phase study the usefulness and feasibility of app utilization in clinical care. Predictive analytic models are built, trained, and selected, and make use of machine learning methods. Models use sensor and phone utilization data to predict behavioral changes and symptoms.

**Results:**

The study started in October 2020. It has received institutional review board approval. The user-centered design phase, consisting of focus groups, usability testing, and preintervention clinician interviews, was completed in June 2021. Recruitment and enrollment for the mobile sensing phase began in October 2021.

**Conclusions:**

Findings may inform the development of passive sensing apps and self-tracking in patients with SMI, and integration into care to improve assessment, treatment, and patient outcomes.

**Trial Registration:**

ClinicalTrials.gov NCT05023252; https://clinicaltrials.gov/ct2/show/NCT05023252

**International Registered Report Identifier (IRRID):**

DERR1-10.2196/39010

## Introduction

### Background

Serious mental illnesses (SMI), such as schizophrenia and bipolar disorder, are disabling and intrusive disorders that can impact educational attainment, work productivity, social functioning, and life expectancy [1]. Psychosocial and psychopharmacological interventions can improve symptoms and health, employment, and education [2]. For individuals with schizophrenia, maintenance antipsychotic treatment is the primary approach for treatment; however, on average, 42% of patients with schizophrenia have been found to be nonadherent to medication treatment, often resulting in worse symptomatology [3,4]. Worsening symptoms can lead to decreased social interaction and make patients less likely to seek help [5,6]. Thus, patient symptoms can worsen or relapse while presenting limited opportunities for mental health clinician awareness, and little opportunity to intervene.

Although warning signs for symptom exacerbation or relapse differ among individuals, they are often consistent within an individual over time. Exacerbations start as mild and increase as symptoms worsen, with 75% of families reporting observable changes between 2-4 weeks before relapse [7]. A study found that the interval between worsening symptoms and relapse was at least one week in 50% of patients [8]. Given a window of opportunity to intervene, interventions were developed and tested to prevent relapse or hospitalization through intermittent drug techniques; however, these interventions showed modest to no effectiveness when implemented [4]. There was noted difficulty getting individuals to reliably self-monitor and report their warning signs early enough for medication changes to prevent relapse, suggesting a need for additional patient support and more reliable monitoring.

Mobile apps and passive mobile sensing technologies could be an approach to help people with SMI self-monitor [9]. Passive mobile sensing can use sensor and phone data from mobile devices to detect health-related behaviors (eg, exercise, social interaction, sleep), which may allow one to detect changes in symptoms and functioning across various behavioral domains like activity, sociability, and sleep [10,11]. These associations have been found in patients with SMI. For example, behavioral indicators for psychiatric symptoms of mood and anxiety disorders monitored through mobile sensing were found to predict clinically assessed symptoms of depression and posttraumatic stress disorder [12]. In patients with bipolar disorder, depressive and manic symptoms were associated with changes in activity and phone communication, while other studies found activity and location to be correlated with mood states [13-16]. In patients with schizophrenia, self-reported stress, depression, and psychotic experiences were also associated with mobile sensor data related to sleep, activity, and communication, signaling the potential for developing predictive models from passive mobile sensing data to aid in identifying changes in symptoms and functioning [17].

Approximately 75% of individuals in treatment for SMI report being willing to participate in digital interventions for stress, health, and mental health [18]. Additionally, patients with SMI find computerized self-assessments acceptable provided they have a clinical application and can help prevent serious adverse events for themselves or others. In a previous study of patients with schizophrenia, passive mobile sensing was found feasible when participation was voluntary [19]. This suggests at least a degree of acceptability among patients with SMI to use passive mobile sensing for managing SMI symptomatology.

Although a few previous mobile sensing studies have centered on individuals with SMI, very few have focused on US veterans—a population with unique physical and mental health needs that may also benefit from this technology. Approximately 4% of veterans have SMI and an estimated 44% of veterans with SMI have moderately severe or severe symptoms [20]. According to a review of health care utilization by veterans, it was found that veterans with SMI were the highest users of inpatient services, with 22% and 41% of patients requiring hospitalization or an emergency department visit, respectively [21]. There are significant effects on patients with SMI and their families associated with worsening symptoms that can lead to diminishing quality of life and medical health. Relative to patients with other mental illnesses, patients with SMI had the greatest percentage of deaths during a 1-year follow-up period [21]. The human costs associated with relapse in patients with SMI are significant, and there is a need to address the difficulty with getting patients to reliably self-monitor and promptly report their warning signs.

A 2014 Veterans Affairs study found that 60% of 249 patients with SMI had a smartphone, with 64% of smartphone users also using apps on their phones [22,23]. Veterans with SMI used similar smartphone functions as the general population and typically kept their phones with them most or all of the time, suggesting that passive mobile sensing may be feasible for this population [22]. Additionally, as time is required for the detection of worsening symptoms and intervention, the period between warning signs and potential relapse is noteworthy. Veterans with SMI on average experienced early warning signs of worsening symptoms 208 days prior to relapse, similar to patients in the general population with SMI [24]. Considering smartphone usage and period of time prior to relapse, passive mobile sensing may be a viable means for patients with SMI to improve outcomes through the development of self-monitoring tools that can help detect or predict worsening symptoms, allowing for earlier assessment, intervention, and improved outcomes.

### Research Aims and Objectives

This study aims to determine the feasibility, safety, and acceptability of passive mobile sensing in patients in treatment for SMI, and to develop and study predictive analytic models to identify associations between symptoms and behavioral domains measured.

The objectives are as follows:

1. Using focus groups and usability testing, conduct user-centered design of passive mobile sensing with self-tracking of activities, sociability, and sleep
2. Study the feasibility, acceptability, and safety of passive mobile sensing of mental health with feedback of mental health status to participants
3. Use mobile sensor and phone utilization data to develop individualized estimates of sociability, activities, and sleep as measured through weekly interviews
4. Study the predictive value of using data on sociability, activities, and sleep to identify worsening of psychiatric symptoms

## Methods

### Study Design

**Figure 1:**
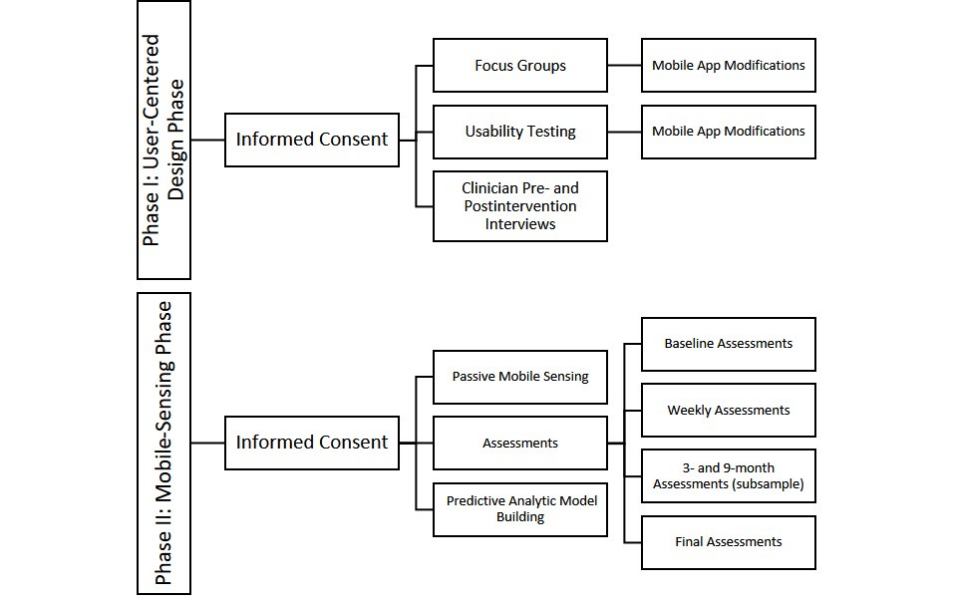
Study phases: user-centered design and mobile-sensing phase.

The study consists of two phases: a user-centered design phase and a mobile sensing clinical trial phase (Figure 1). The study uses a passive mobile sensing app that displays data gathered in the form of interactive patient dashboards. The user-centered design phase (Phase I) focuses on usability of and perceptions regarding the app. The mobile sensing phase (Phase II) studies the safety, feasibility, and acceptability of passive mobile sensing, and uses data to develop predictive models for behavioral domains associated with SMI symptoms.

### Eligibility and Screening

All participants are recruited from the VA Greater Los Angeles Healthcare System (GLA VA), with clinicians included if they are providing care for veterans with SMI. The following inclusion criteria are used: (1) clinical diagnosis of SMI, defined as schizophrenia, schizoaffective disorder, or bipolar disorder, (2) risk for symptoms based on having had, during the past year, psychiatric hospitalization, psychiatric emergency care, lived at a crisis program, or more than 6 outpatient visits, and (3) ownership of a phone running Android OS with a data plan. The following exclusion criteria are used: (1) under the age of 18 years and (2) has a conservator/legally authorized representative.

### Study Site and Recruitment of Study Participants

A list of potentially eligible participants was identified using Veterans Affairs data systems. In addition, flyers were posted at clinics and announcements were made at group meetings to invite participation. Individuals interested in participating had a phone screening verifying diagnosis, age, and last treatment. After confirming potential eligibility, prospective participants were scheduled for an in-person or virtual screening visit at which further information was provided and eligibility assessments were re-verified. Additionally, individuals were asked if they own a smartphone running Android OS with a data plan. If an individual was eligible, full informed consent was obtained, and they were enrolled.

### Ethics Approval

The study has been approved by the Institutional Review Board of the GLA VA (project number IIR 19-392).

### Phase I: User-Centered Design

#### Data Collection

**Figure 2:**
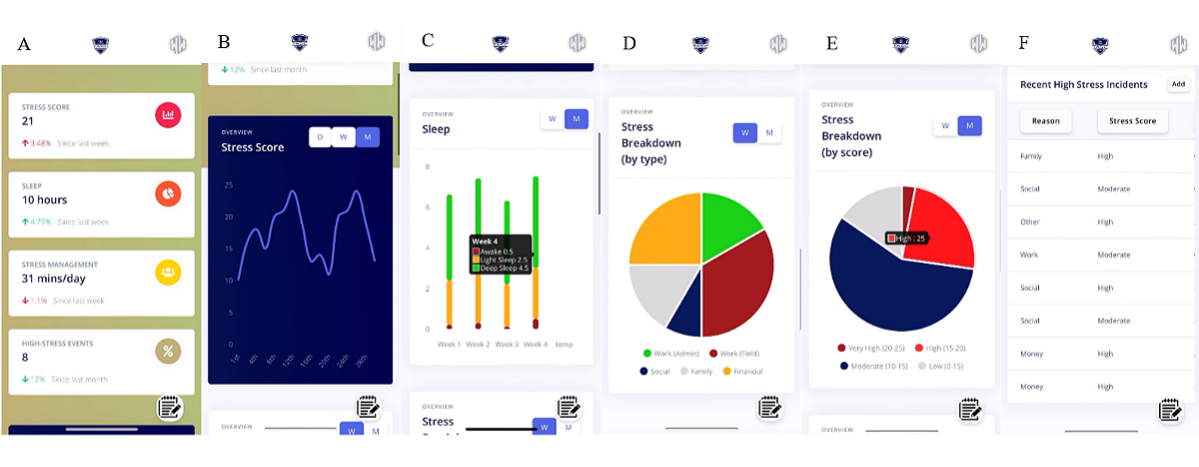
Mobile self-tracking app dashboard prototype examples. (A) Overview of behaviors. (B) Interactive score calculated over time. (C) Chart breaking down behavioral details over time. (D) Chart breaking down behaviors into categories. (E) Chart breaking down scores over a week or month on the scale of low, moderate, high, and very high. (F) Notes on recent incidents and their severity. Higher-resolution version of this figure is available in Multimedia Appendix 1.

To study the feasibility, acceptability, and safety of passive sensing and self-tracking, the Technology Acceptance Model was used [25]. Components of the Technology Acceptance Model include perceived usefulness, ease of use, attitudes, self-efficacy, norms, facilitating conditions, and behavioral intent. Three focus groups and usability testing were conducted to receive feedback regarding the app and new dashboards. The interactive dashboards for activities, sleep, and sociability display scores by type and intensity, as well as over time (Figure 2A-Figure 2F, Multimedia Appendix 1). Focus groups were co-led by a qualitative methods expert and investigator, with each group containing six veterans. Participants of focus groups were asked questions regarding perceived usefulness, ease of use, attitudes, self-efficacy, norms, facilitating conditions, and behavioral intent. Focus groups aimed to elucidate the acceptance and usability of the dashboards and the app overall.

Modifications were made in response to focus group feedback and usability trials. Usability trials were conducted by a qualitative methods expert and research assistant with 8 veterans meeting inclusion criteria. Veterans participated individually and were asked to complete specific tasks on the app (eg, find the sleep dashboard). They were asked about downloading and opening the app, reviewing dashboards, closing the app, and managing the app. They were asked to express their thoughts as they navigated the app, and RAs noted what the participants said and did. Both focus groups and usability testing lasted approximately 45 minutes.

Before and after the trial phase, semistructured interviews were conducted with clinicians who provide treatment to patients with SMI at the GLA VA. Preintervention interviews with clinicians assessed the acceptability of mobile sensing, its usefulness as a tool to improve clinical assessment and care, and recommendations for improvement. Post–sensing phase interviews will assess how to engage patients, and reflect on findings, implementation issues, and resources needed for sustainability and incorporation into routine practice.

#### App Development and Maintenance

Modifications to the app are rolled out in response to information security requirements, operating system updates, characteristics of phone devices, and needed changes for data acquisition or transmission. Qualitative data collected during user-centered design, as well as data from the mobile self-tracking phase, are used to guide modifications. Some modifications may require a new download of the app, which can be done using phone-based software or during weekly research assessment calls.

### Phase II: Mobile Sensing

Following consent and enrollment, participants underwent a baseline assessment. This consisted of an interview regarding demographics, symptoms, functioning, treatment utilization, mobile phone usage, housing status, substance abuse, and cognitive deficits (Table 1). Additionally, throughout the sensing phase of the study, weekly assessments were conducted by phone with participants to assess their sleep, sociability, activities, symptoms, and safety. The rate of serious adverse events during participation will be monitored.

**Table 1:** Overview of data collection instruments and schedule of assessments for passive mobile sensing phase.

Measure	Data collected and measuring instrument	Visit administered
Sociodemographic data	Gender, ethnicity, race, marriage, work and school history, service history	Baseline, final
Psychiatric illness history	The World Health Organization Disability Assessment Schedule (WHODAS 2.0) [26] Perceived Stress Scale (PSS-4)	Baseline, final
Substance use	DSM-5 Self-Rated Level 1 Cross-Cutting Symptom Measure-Adult^a^	Baseline, final
Cognition	Digit Symbol Coding Test (DSCT)	Baseline, final
Occupation, social, symptom-related, and overall functioning	Mental Illness Research, Education, and Clinical Center Global Assessment of Functioning (MIRECC GAF) [27]	Baseline, final
Medication possession ratio	Pharmacy data 12 months prebaseline [28]	Baseline, final
Health care utilization	Service Use and Resources Form (SURF) [29]	Baseline, final
Housing stability	Residential Time-Line Follow-Back [30]	Baseline, weekly, final
Psychopathology	Brief Psychiatric Rating Scale (BPRS)^b^ [31]	Baseline, weekly, final
Sociability	Abbreviated Lubben Social Network Scale [32] Objective Frequency of Social Contact scale [33,34]	Baseline, weekly, final
Activity, routines, habits	International Physical Activity Questionnaire [35] Social Functioning Scale (SFS)^c^ [36]	Baseline, weekly, final
Sleep	Pittsburgh Sleep Quality Index (PSQI)^d^ [37] Insomnia Severity Index (ISI)^e^ [38]	Baseline, weekly, final

^a^Substance-use items only.

^b^Positive Symptoms Factor (psychosis), Activation Factor (mania), Affect Factor (depression) [39,40].

^c^Independence Performance and Prosocial Domain.

^d^Components 1, 2, 3, and 6.

^e^Insomnia Problem Items.

At the baseline assessment visit, the app was downloaded onto the participant’s phone and activated. Every time the app is activated on a device, an individualized ID is generated by the server. Following activation, the app runs in the background and collects data passively without further action from the user. The server generates an automated report daily that includes the IDs of participants who have not recently transmitted any data, and research staff contact participants to investigate and troubleshoot.

**Figure 3:**
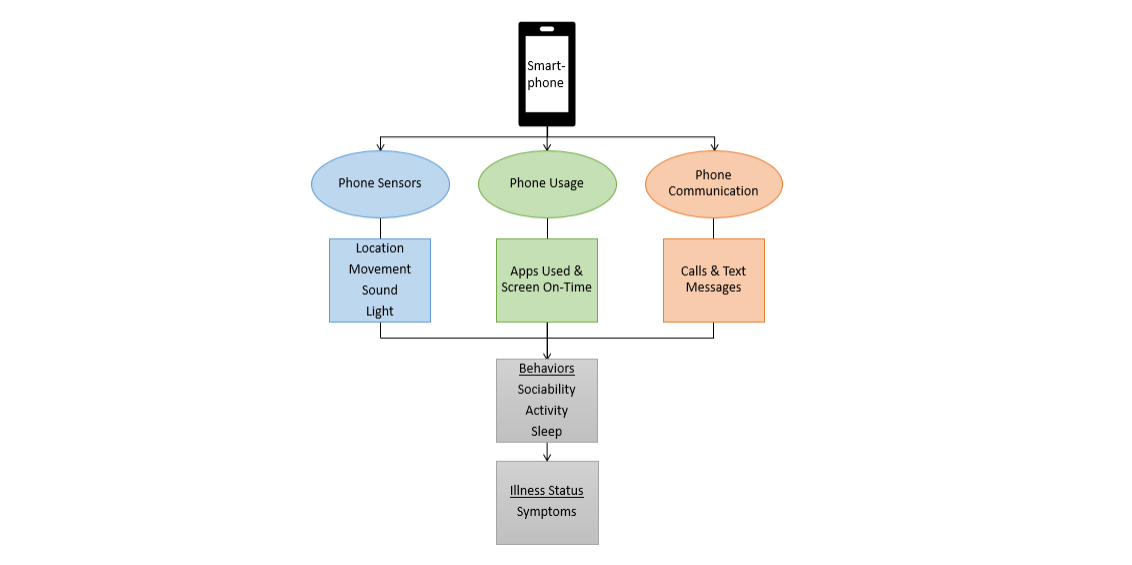
Passive mobile sensing and utilization data and behavioral domains.

The app collects passive behavioral data in three domains that have been previously shown to be measurable with these data: activities, sleep, and sociability (Figure 3). The app uses the AWARE application programming interface (API) that manages the querying and data packaging, transmission, and storage. The input data are drawn from phone utilization, sensors, software on Android phones that process sensor data, and Google APIs (Table 2). The Google Fused Location API and Google Activity Recognition API are used for postprocessing of sensor data. Although most recent phones contain most or all of these data sources, some older phones may lack certain sensors; the app uses all available sensor data.

The data collected from the app are encrypted and cached locally on the device, and transmitted to the platform’s remote server when Wi-Fi is available. If Wi-Fi is not available, data are cached locally until a connection is established. Data are transmitted through an encrypted channel to a web application on a server where it is stored in a secure, encrypted format. Following confirmation of successful transmission, data are deleted from the phone.

A random subsample of participants was selected to partake in 3- and 9-month evaluation assessments, which are semistructured interviews that are recorded and transcribed until data saturation is achieved. Interviews assess for acceptability of passive sensing, usefulness, ease of use, attitudes, and behavioral intention toward use. Participants also provide additional feedback regarding the app. There is a 9-month follow-up and final visit for all participants, in which measures from baseline are readministered.

**Table 2:** Mobile sensing data for modeling of behavioral domains.

Behavioral domain		Input data from phone	Model output classifiers
**Activities**			
	Physical activity intensity and duration vs sedentary Organized activities: number and duration outside the individual’s residence Regular structured activities throughout each day (location and duration)	Accelerometer sensor Linear accelerometer Android software Gyroscope sensor Rotational vector Android software Step counter Android software Significant motion Android software Activity Recognition Google API^a^ Fused Location Google API (uses GPS and Wi-Fi)	Short International Physical Activity Questionnaire (4 items) [35] Independence, Performance and Prosocial domains of the Social Functioning Scale (15 items) [36]
**Sleep**			
	Total sleep duration Uninterrupted sleep Regular daily sleep and wake times	Ambient light sensor Ambient sound sensor Significant motion Android software Fused Location Google API (uses GPS and Wi-Fi) Phone unlock, screen interactions, and on-time duration Log of phone calls placed Log of messages sent Apps opened App duration of use	Pittsburgh Sleep Quality Index Components 1, 2, 3, 6 (5 items) [37] Insomnia Problem items from the Insomnia Severity Index (3 items) [38]
**Sociability**			
	Communication in person or in a public social environment Communication with a diverse set of individuals Communication with repeated partners	Log of phone calls placed with phone numbers Log of phone calls received with phone numbers Log of messages sent with phone numbers Log of messages received with phone numbers Social media apps opened Social media apps (keystrokes and duration used) Messaging or email apps opened Messaging or email apps (keystrokes and duration used) Ambient sound sensor Activity Recognition Google API used Location Google API (uses GPS and Wi-Fi)	Abbreviated Lubben Social Network Scale (6 items) [32] Objective Frequency of Social Contact scale (6 items) [34]

^a^API: application programming interface.

### Data Management and Security

During informed consent, participants are informed about how the system works; how data are collected, transmitted, and stored; and who has access to the data. The mobile app communicates with a secure server where sensor data are housed. Data are encrypted when at rest on the mobile device, in transit to the server, and on the server.

### Data Analysis Plan

#### Feasibility, Acceptability, and Safety

Qualitative interviews are professionally transcribed and analyzed deductively for major subthemes of usefulness, ease of use, attitudes and behavioral intention toward use, acceptability, strengths and weaknesses, barriers and facilitators of use, safety, and recommendations about revisions and implementation efforts. Results inform adjustments of the app and modifications of methods for enrollment and maintenance of participation.

Data obtained from the mobile self-tracking phase’s quantitative baseline patient characteristics will be studied using multivariate analyses to determine potential associations with the themes of feasibility, acceptability, and safety of passive sensing. Qualitative data from the 3-month and 9-month interviews will be used to evaluate these themes.

Feasibility is characterized by studying the extent to which potential subjects enroll, maintain involvement, and complete study assessments. Acceptability is characterized by counting the number of days participants use mobile sensing and dashboards, as well as by analyzing data from semistructured interviews. Safety is measured using the rate of serious adverse events. Additionally, important medical events may also be considered a severe adverse event if jeopardizing the participant or requiring a medical or surgical intervention.

#### Building Predictive Models

Mobile phone sensor and utilization data are used to develop individualized estimates of sociability, activity, and sleep that will also be measured through weekly interview. Data gathered by the app provide detailed patient data over the follow-up period. Real-time data streams construct behavioral measures of interest. As data are collected at vastly different frequencies and durations with different noise and error rates, a 2-step modeling approach is used. Step 1 preprocesses sensing data to derive features capturing events of interest. Step 2 builds prediction models using machine learning techniques to estimate correlations between sensor and phone utilization data, and behavioral assessments and symptoms.

During the data preprocessing step (Step 1), sensor and utilization data are captured at varying frequencies for different domains and summarized using activity profiles (over a day or a week). Daily or weekly activity profiles are constructed based on the amount of time that the individual spends at an activity level (eg, above a threshold) or engaged in an activity at a given day or week.

In Step 2 (prediction model development), various machine learning algorithms are used to build, train, and select prediction models for each of the patient’s behavioral assessment domains. Model development occurs in three stages: unsupervised learning to reduce dimensionality (Stage 1), supervised learning to build candidate models (Stage 2), and cross-validation to estimate out-of-sample performance of the prediction model and select the best models (Stage 3). Models developed through supervised machine learning methods are evaluated in the out-of-sample predictive performance using leave-one-out cross-validation.

## Results

The study started in October 2020. The patient focus groups, usability trials, and clinician interviews enrolled 17 veterans, 8 veterans, and 16 clinicians, respectively. Data collection for the user-centered design phase was completed in June 2021. Recruitment for the mobile tracking phase started in October 2021 with the goal of recruiting 125 patients. This study is expected to conclude in July 2023.

## Discussion

### Overview

This study will determine the feasibility and acceptability of passive mobile sensing, and estimate the extent to which behavioral data predicts behaviors and psychiatric symptoms in patients with SMI. We anticipate that passive mobile sensing will be feasible and acceptable in most patients with SMI. We hypothesize that passive mobile data will be associated with behaviors and psychiatric symptoms, and that these associations will vary among individuals.

Passive mobile sensing could be an innovative method to improve self-monitoring of behaviors associated with worsening symptoms of SMI. There is increasing support for the validity of behavioral indicators for mobile sensing platforms as predictive of psychiatric symptoms [12,41]. Additionally, the feasibility and acceptability of passive mobile sensing in patients with SMI has been explored with encouraging results [41,42].

### Potential Limitations

Some challenges are expected in recruitment, enrollment, and retention in the patient population. To overcome these difficulties, strategies like monitoring and tracking patient flow, fair and appropriate compensation, and flexible assessment scheduling are used. Due to the COVID-19 pandemic, it has been difficult to conduct in-person screening visits; consequently, the study is adopting virtual screening, consents, and assessments to recruit and enroll participants.

Additionally, as the data are collected from smartphones, we expect missing data points. Various approaches will be considered to adjust for missing data when creating activity profiles. One such approach is the similarity assumption that the proportion of time that a participant spends at a given activity level during observed times is like that during unobserved times, which is similar to the missing at random assumption in longitudinal data analysis. Another approach is to use the planned machine learning approaches to evaluate missing data patterns.

### Conclusion

If found to be feasible, acceptable, and safe, passive mobile sensing can become a clinical tool for patients with SMI, allowing access to dashboards of activity, sociability, and sleep that can be used as a tool for monitoring symptoms and behaviors. The adoption of such apps may also be viable and its integration into clinical care may further improve clinical outcomes for patients with SMI. The findings of this study hope to guide the development of passive sensing and modeling that could dynamically assess mental health and identify the risk for worsening illness. It hopes to eventually guide the development of a platform that is acceptable and desirable to patients, helping patients and their clinicians to monitor their clinical status, identify the risk for relapse, and allow for early intervention.

## Appendix

Multimedia Appendix 1 Higher resolution version of Figure 2. Mobile self-tracking app dashboard prototype examples. (A) Overview of behaviors. (B) Interactive score calculated over time. (C) Chart breaking down behavioral details over time. (D) Chart breaking down behaviors into categories. (E) Chart breaking down scores over a week or month on the scale of low, moderate, high, and very high. (F) Notes on recent incidents and their severity.

Multimedia Appendix 2 Peer-reviewer report from the HSR-4 Mental and Behavioral Health - Health Services Research Parent IRG - Office of Research & Development.

## Abbreviations

API: application programming interface
GLA VA: VA Greater Los Angeles Healthcare System
SMI: serious mental illness

## Data Availability

Mobile sensor data collected during this project contain information that is sensitive. These data are also difficult to deidentify to a level that ensures that data cannot be reidentified in the future. As a result, data will not be available in public repositories and access to data is restricted.
