# Supplementary figures and images for: Passive Mobile Self-tracking of Mental Health by Veterans With Serious Mental Illness: Protocol for a User-Centered Design and Prospective Cohort Study
Source: JMIR Res Protoc. 2022 Aug 5;11(8):e39010. doi: 10.2196/39010 (PMC9391975; doi:10.2196/39010)

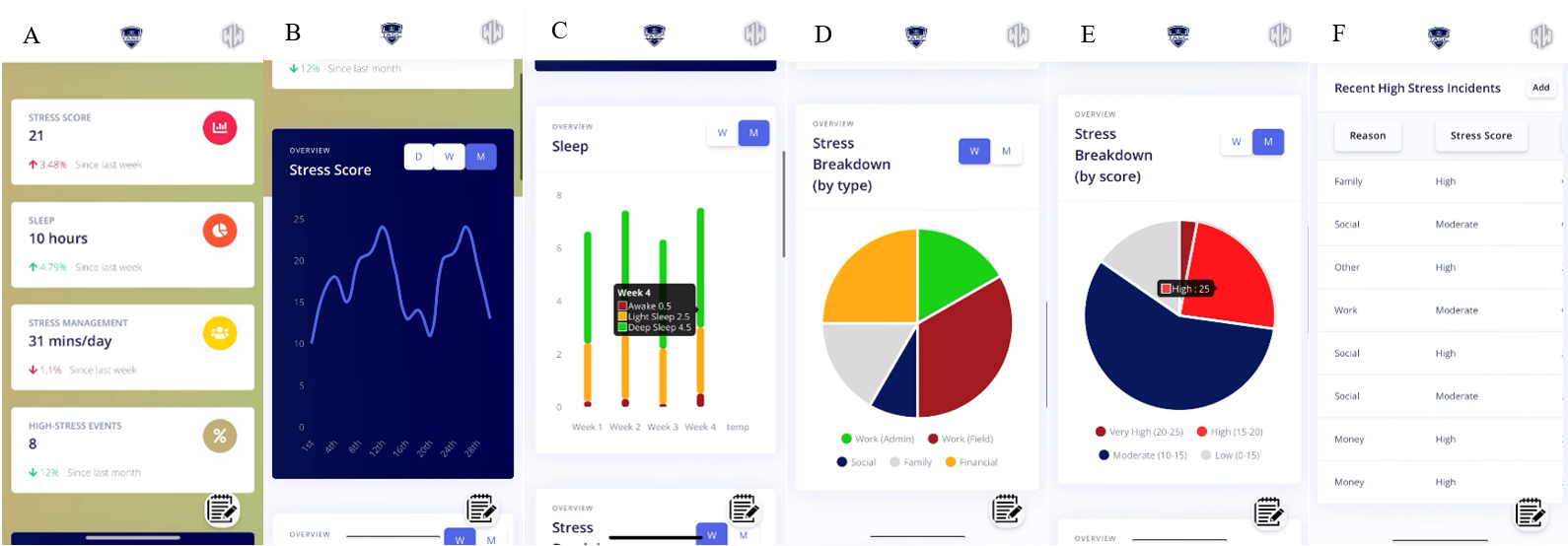

Supplement: Multimedia Appendix 1 [file resprot_v11i8e39010_app1.png]
