# Supplementary material for: Passive Mobile Self-tracking of Mental Health by Veterans With Serious Mental Illness: Protocol for a User-Centered Design and Prospective Cohort Study
Source: JMIR Res Protoc. 2022 Aug 5;11(8):e39010. doi: 10.2196/39010 (PMC9391975; doi:10.2196/39010)
Supplement: Multimedia Appendix 2 [file resprot_v11i8e39010_app2.pdf]

SUMMARY STATEMENT

PROGRAM CONTACT: **(Privileged Communication)** ***Release Date:*** **04/02/2020**

Revised Date:

Application Number: **1 I01 HX003129-01A1**

Principal Investigator

YOUNG, ALEXANDER S.

Applicant Organization: VA GREATER LOS ANGELS HEALTHCARE SYSTEM

Review Group: **HSR4**
HSR-4 Mental and Behavioral Health

Meeting Date: **03/05/2020** ***RFA/PA:*** **HX20-001**
Council: **MAY 2020** ***PCC:*** **HXOBRI**
Requested Start: **07/01/2020**

Project Title: **Passive Mobile Self-Tracking of Mental Health by Veterans with Serious Mental Illness**
SRG Action: **Impact Score:130 Percentile:3.0 +**

Human Subjects: **20-Human subjects involved - No exemption designated**
Animal Subjects: **10-No live vertebrate animals involved for competing appl.**

Project Year	Direct Costs Requested
1	281,036
2	292,302
3	202,806
TOTAL	776,144

ADMINISTRATIVE BUDGET NOTE: The budget shown is the requested budget and has not been adjusted to reflect any recommendations made by reviewers. If an award is planned, the costs will be calculated by VA Office of Research and Development (ORD) staff based on the recommendations outlined in the BUDGET COMMENT section and any relevant ORD service-specific limitations.

KEY SUMMARY POINTS:

1. Very responsive to previous comments; strong VA program support; strong team; study has potential to be highly significant given VA investment in this population
2. Study is very innovative, particularly with inclusion of artificial intelligence and passive data will be made available interactively to Veterans
3. Measurement of provider use of data in treatment is underdeveloped

DESCRIPTION (provided by applicant):

Background: Serious mental illnesses are common, disabling, challenging to treat, and require years of monitoring with adjustments in treatments. Stress or reduced medication adherence can lead to rapid worsening in symptoms and functioning with consequences that include relapse, job loss, homelessness, incarceration, hospitalization and suicide. In usual care, clinician visits are infrequent, with intervals ranging from monthly to yearly. Communication between patients and clinicians between visits is challenging and often nonexistent. Patient illness exacerbations and relapses generally occur with little or no clinician awareness in real time, leaving little opportunity to adjust treatments. Significance/Impact: For the large population of Veterans with serious mental illness, tools are needed that passively monitor their mental health status, allowing them to self-track their behaviors, quickly detect worsening of mental health, and support prompt assessment and intervention. At least 60% of Veterans with serious mental illness use a smart phone. These generate data that characterize sociability, activity, and sleep. Changes in these behaviors are warning signs of relapse. Passive self-tracking could be used to identify and predict worsening of illness in real time. Innovation: Passive mobile sensing is a novel approach to illness self-tracking and monitoring. There has been relatively little research on passive self-tracking in serious mental illness, with limited analytics development in this area, and none in VA. Specific Aims: This project studies passive mobile sensing with Veterans in treatment for serious mental illness. Data are used for self-tracking of behaviors and symptoms. While passive mobile sensing has been feasible, acceptable and safe in patients with serious mental illness, these are studied for the first time in VA. Analytics are developed that use passive data to predict behaviors and symptoms. This project responds to the HSR&D priority areas of Mental Health and Healthcare Informatics. The project has these objectives: 1. Conduct user-centered design of passive mobile self-tracking to support Veterans' management of their mental health. 2. Study the feasibility, acceptability and safety of passive self-tracking of mental health that includes feedback of mental health status to the Veteran. 3. Use mobile sensor and phone utilization data to develop individualized estimates of sociability, activities, and sleep as measured by weekly interviews. 4. Study the predictive value of using data on sociability, activities, and sleep to identify exacerbations of psychiatric symptoms. Methodology: Activities can be assessed with data on movement, location, and habits. Sociability can be assessed with data on communication and public interactions. Sleep can be assessed using data on light, sound, movement, and phone use. Investigators on this project developed "mWellness," a functional mobile app that monitors and transmits mobile sensor and utilization data. Focus groups and in-lab usability testing will inform further app and intervention development. Mixed methods research will study deployment in Veterans who passively self-track their behaviors and psychiatric symptoms. If this project meets intended goals, the VA will have a mobile analytics platform that continuously monitors the behaviors and symptoms of patients with serious mental illness. Next Steps/Implementation: Results inform development of a study of the effectiveness and implementation of these methods to improve Veteran assessment, treatment and outcomes. Results are also applicable to other populations with mental health risk and to the use of similar methods in usual care.

CRITIQUE 1

1. Significance.

The study evaluates passive mobile sensing with Veterans in treatment for serious mental illness and allows self-tracking of behaviors and symptoms. Analytics are developed that use passive data to predict behaviors and symptoms. The investigators plan to:

1. Conduct user-centered design of passive mobile self-tracking to support Veterans' management of their mental health.
2. Study the feasibility, acceptability and safety of passive self-tracking of mental health that includes feedback of mental health status to the Veteran.
3. Use mobile sensor and phone utilization data to develop individualized estimates of sociability, activities, and sleep as measured by weekly interviews.
4. Study the predictive value of using data on sociability, activities, and sleep to identify exacerbations of psychiatric symptoms.

The study has substantial significance in that technology could improve the ability of patients, families, and providers to have earlier warning signs of relapse of serious mental illnesses that involve psychotic, manic, or suicidal episodes. Warning signs of relapse include changes in sleep patterns and social patterns (i.e. either social isolation or overactivity). The investigators propose that smartphone application for the passive collection of sensor data can be used to help identify risk for relapse. The advancement in science would be meaningful and large enough to warrant a study such as the one proposed.

2. Innovation and Impact.

The investigators have developed new technology for a smartphone application that may have important impact in self- management of illness. This project would yield results that could be expected to change current research and clinical practice paradigms. This technology has not been tested in a VA setting/population. This tool would be highly innovative and has potential for clinically meaningful impact. The technology could also be very useful for other research to track outcome to interventions.

3. Approach.

Approach as written in the application:

Methodology: Activities can be assessed with data on movement, location, and habits. Sociability can be assessed with data on communication and public interactions. Sleep can be assessed using data on light, sound, movement, and phone use.

Investigators on this project developed “mWellness,” a functional mobile app that monitors and transmits mobile sensor and utilization data. Focus groups and in-lab usability testing will inform further app and intervention development. Mixed methods research will study deployment over 9 months in 60 Veterans who passively self-track their behaviors and psychiatric symptoms. If this project meets intended goals, the VA will have a mobile analytics platform that continuously monitors the behaviors and symptoms of patients with serious mental illness.

Key summary points in the previous critique and key modifications:

- More clarity needed regarding primary outcome of exacerbated symptoms Added the Brief Psychiatric Rating Scale and updated the analysis plan.
- Heterogeneity of diagnosis is not sufficiently addressed.
- Excluding MDD with psychotic features; narrowing to schizophrenia and bipolar.

- Unclear how this app would be integrated into VA care.

Processes could be automated using the VA Office of Connected Care patient-generated data platform that currently houses video telehealth and other Veteran data to interface with clinicians. No longer needs to connect within VA firewall (i.e. Video Connect and MyHealtheVet). Included a support letter from VA OCC. VA OCC and VA Office of Mental Health and Suicide Prevention provided support letters to sustain the app and future implementation study would be proposed.

- Missed opportunity to collect perspectives of VA providers.
- Added individual interviews with clinicians and qualitative analyses to inform development.

Other changes to enhance the approach:

- Increased sample size from 60 to 100 and extended recruitment period.
- Increase timeline for initial aim from 4 to 6 months to allow interview with providers.
- Added a process for communicating risk of clinical deterioration to patient’s clinician.

4. Feasibility (including Sampling, Project Timeline and Staffing).

Increasing the sample size from 60 to 100 does not negatively impact feasibility. They will be recruiting from a very large patient pool in the Greater LA VA Healthcare System. The investigators have been thoughtful in modifying the timeline due to increased activities in Aim 1 and in recruitment goals.

5. Implementation.

The investigators have already thought through the logistics of taking the product from test phase to implementation phase and have garnered the support of VA OCC and VA OMHSP.

6. Investigator Qualifications.

The investigators are experienced, qualified and capable. They have a track record of success.

7. Multiple PI Leadership Plan.

Not applicable.

8. Facilities and Resources.

The facilities and resources are well established and robust. The investigators have support at the national level.

9. Adequacy of Response to Previous Feedback Regarding the Proposed Study.

The investigators have responded to all points and feedback from previous review.

10. Protection of Human Subjects from Research Risk.

The investigators have provided reassurance at multiple levels that privacy risks are minimal.

11. Inclusion of Women and Minorities in Research.

Women and minorities will be included.

12. Budget and Period of Support.

The study has been modified to a 3 year study (from 2 years) and costs are appropriately increased to $776,144.

13. Sharing Research Data (Data Management and Access Plan (DMAP)).

The investigators plan to make de-identified datasets available under appropriate approvals and data use agreements.

14. Overall Impression.

The technology is innovative, clinically meaningful, and needed to fill a gap. The research methods are well described and thorough. The investigators have been responsive to previous review critique.

15. Key Strengths.

1. The study is focused on finding clinically meaningful self-management tools that can help a manage a difficult-to-treat patient population.

2. Innovative technology and support from VA OCC to make it a reality.

3. Responsive to reviewers' critique.

4. Very strong investigative team and environment.

5. Letters of support from VA OCC and VA OMHSP.

16. Key Weaknesses.

1. The test of predictability of self-tracking symptoms given many confounding variables is always something of concern with innovative research; not a weakness necessarily - just a reality.

CRITIQUE 2

1. Significance.

Understanding how mobile technology can be used to assist individuals with Severe Mental Illness (SMI) as well as others with mental illness is a pressing question. While many individuals will opt out of anything requiring passive tracking, many are willing. This proposal to develop and evaluate the acceptability, feasibility, and validity of passive sensing for prevention of decompensation in SMI is conceptually thoughtful and interesting. The idea of making the passive sensing results visible to the Veteran is particularly interesting as seeing what is being analyzed may increase the comfort of the Veteran to participate as well as providing self-monitoring benefits. The VA is a large, complex, and politically sensitive system which all can lead to barriers to technological interventions. Thus, it is critical that health service technological interventions be developed to effectively co-exist in VA care. VA partners have provided letters supporting their investment. If they can, this will be extremely significant.

2. Innovation and Impact.

Previous SMI mobile phone interventions have required significant attention from the individual to function properly and many individuals with SMI have other concerns and did not interact sufficiently – limiting the benefit of these mobile apps. This study is innovative in focusing purely on the passive sensing and demanding nothing else from the Veteran with SMI while offering self-monitoring information from the passive sensors, should the Veteran care to view it. Given the operational support for integrating the app into VA care, the impact could be very large.

3. Approach.

This is a well-written grant by experienced researchers who clearly describe a reasonable set of methods to match their aims which build one upon the next. Starting with user-centered design and in-lab testing will be critical. Their plan to parallel the passive sensing data with ongoing reports from the participants on standard measures will provide key information on the validity and utility of the passive sensing data compared to gold standard measures of mechanisms and outcomes.

4. Feasibility (including Sampling, Project Timeline and Staffing).

The project team provides sufficient detail with regard to the recruitment and staffing. The revised proposal adds sample size and time to the project that will increase feasibility.

5. Implementation.

The investigators have expanded their implementation plan through greater support from operational partners and more explicit next steps.

6. Investigator Qualifications.

Excellent.

7. Multiple PI Leadership Plan.

Not applicable.

8. Facilities and Resources.

More than adequate.

9. Adequacy of Response to Previous Feedback Regarding the Proposed Study.

Not applicable.

10. Protection of Human Subjects from Research Risk.

Adequate.

11. Inclusion of Women and Minorities in Research.

Adequate.

12. Budget and Period of Support.

Adequate.

13. Sharing Research Data (Data Management and Access Plan (DMAP)).

Adequate.

14. Overall Impression.

This is a promising project to develop a passive sensing app that could be alert to early signs of decompensation in Veterans with SMI. The research team has a thoughtful and systematic approach laid out that reflects appropriate treatment development methodology. The ability for Veterans to review their own data is innovative and potentially impactful.

15. Key Strengths.

1. Could play a useful role in predicting decompensation allowing for timely intervention.
2. Strong research team.
3. Strong treatment development methodology.
4. User-centered design principles.

16. Key Weaknesses.

1. Presuming the VA systems will allow for future compatibility.

CRITIQUE 3

1. Significance.

The proposed research has very high public health significance. Ten percent of VHA's budget is allocated to the care of Veterans with SMI.

The proposed research targets two HSR&D research priorities (Mental Health and Healthcare Informatics).

The background of the application makes a solid justification for the use of passive monitoring to detect relapse, which is quite common among patients with SMI. A passive sensing approach is needed because interventions focused on active self-monitoring have proven to be ineffective.

2. Innovation and Impact.

The proposed use of passive monitoring is highly innovative.

The proposed use of AI to analyze the passive monitoring and weekly symptom ratings is highly innovative.

The use of user centered design methods to improve patient-centeredness is novel.

There are no overlapping ongoing studies funded by VA or other federal research agencies.

3. Approach.

User Centered Design methods are proposed, which should make the mWellness highly Veteran centric.

Use of the Technology Acceptance Model to guide Aim 2 is a strength.

The application provides a detailed description of the app, with special attention to the potential risks to Veterans (which are minimal). The evaluation will assess app functioning and acceptability (data collection/transmission) as well as Veterans' perceptions of the app.

The application provides considerable detail about how the passive data will be collected and analyzed, including how to manage sensors collecting data at different time frequencies, data reduction techniques, and missing data.

A minor weakness of the proposed methods is that there is no quantitative assessment of how the clinicians view the alerts. Will the clinicians react to the alerts (i.e., are they clinically actionable)? Will new appointments be scheduled for patients with alerts? These questions will be assessed qualitatively, which mitigates this weakness somewhat.

Another weakness is that the application lacked detail on establishing baseline behaviors and whether Veterans would be recruited in a "baseline" state or a "relapsed" state. It was not clear whether the AI could handle data from patients recruited when they were in a relapse state?

Finally, while the addition of the self-monitoring dashboard to the mWellness app is a strength, the evaluation plan did not really specify how the investigators would determine whether this additional feature contributes to improved outcomes. The perceived usefulness of dashboard will be assessed qualitatively/retrospectively, which mitigates this weakness somewhat.

4. Feasibility (including Sampling, Project Timeline and Staffing).

A high percentage of Veterans with SMI use smartphone functionality. A small pilot with UCLA students found no major technical problems with the mWellness app. The investigators have already obtained approval for a mobile phone sensing and predictive analytic research project from their VA IRB, Information Security Officer, and R&D service. There are more than a sufficient number of Veterans with SMI at the GLA VAMC to recruit the proposed sample.

5. Implementation.

The D&I plan is likely to be effect. The research is strongly supported by the Office of Connected Care.

6. Investigator Qualifications.

Dr. Young is at the forefront of research in mHealth for Veterans with SMI and has been a highly productive investigator over the years. He is proposing to devote a substantial amount of FTE to the project, ensuring its successful completion.

There was obviously substantial involvement from Dr. Majid Sarrafzadeh (AI expert on the team) in the development of the application. Involving computer science experts from UCLA in the research project is a major strength of the application.

7. Multiple PI Leadership Plan.

Not applicable.

8. Facilities and Resources.

Research resources at the GLA VAMC (HSR&D COIN, MIRECC, UCLA affiliation) are outstanding.

9. Adequacy of Response to Previous Feedback Regarding the Proposed Study.

The investigators have been highly responsive to the previous critiques. The application is much improved.

10. Protection of Human Subjects from Research Risk.

Adequate.

11. Inclusion of Women and Minorities in Research.

Adequate.

12. Budget and Period of Support.

Acceptable.

13. Sharing Research Data (Data Management and Access Plan (DMAP)).

Acceptable.

14. Overall Impression.

The proposed research addresses a disorder that consumers 10% of VHA funding and addresses two key HSR&D research priorities. This highly innovative research is being proposed by an outstanding investigative team that includes both VA and UCLA researchers. The main concern about the proposed research (fidelity) has been very well addressed. The approach had a few minor/negligible weaknesses.

15. Key Strengths.

1. The proposed research has high public health significance and addresses two HSR&D research priorities.

2. The passive monitoring approach is well justified.

3. The user centered design and AI methods are highly innovative.

4. The investigator team is exceedingly strong.

5. Feasibility issues were thoroughly addressed.

16. Key Weaknesses.

1. The approach has minor weaknesses with regard to evaluating the contributions of the dashboard, how the AI would handle sensor data from patients in relapse at baseline and determining how clinicians viewed alerts.

Footnotes for 1 I01 HX003129-01A1; PI Name: Young, Alexander S.

+ Derived from the range of percentile values calculated for the study section that reviewed this application.

MEETING ROSTER

HSR-4 Mental and Behavioral Health
Health Services Research Parent IRG
Office of Research & Development
HSR4
03/05/2020 - 03/06/2020

CHAIRPERSON(S)

COHEN, AMY N., PHD
DIRECTOR, IMPLEMENTATION SCIENCE UNIT, VA DESERT PACIFIC MENTAL ILLNESS RESEARCH, EDUCATION, AND CLINICAL CENTER (MIRECC)
GREATER LOS ANGELES VA HEALTHCARE CENTER
ASSOCIATE RESEARCH PSYCHOLOGIST
UCLA CENTER FOR HEALTH SERVICES AND SOCIETY
LOS ANGELES, CA 90073

MEMBERS

BAGGE, COURTNEY L, PHD
PSYCHOLOGIST, RESEARCH INVESTIGATOR
CENTER FOR CLINICAL MANAGEMENT & RESEARCH
ANN ARBOR VA MEDICAL CENTER
ASSOCIATE PROFESSOR
DEPARTMENT OF PSYCHIARTY, UNIVERSITY OF MICHIGAN
ANN ARBOR, MI 48109

BAHRAINI, NAZANIN, PHD
DRIECTOR OF EDUCATION
ROCKY MOUNTAIN MIRECC
ASSOCIATE PROFESSOR, DEPARTMENTS OF PSYCHIATRY AND
PHYSICAL MEDICINE AND REHABILITATION
UNIVERSITY OF COLORADO
AURORA, CO 80045

BELSHER, BRADLEY E, PHD
SECTION CHIEF, RESEARCH TRANSLATION AND INTEGRATION
PSYCHOLOGICAL HEALTH CENTER OF EXCELLENCE
DEFENSE HEALTH AGENCY
ASSISTANT PROFESSOR
DEPARTMENT OF PSYCHIATRY USUHS
SILVER SPRING, MD 20910

BROWN, GREGORY K, PHD
RESEARCH PSYCHOLOGIST
CORPORAL MICHAEL J. CRESCENZ VA MEDICAL CENTER
RESEARCH ASSOCIATE PROFESSOR
PENN PSYCHIATRY
UNIVERSITY OF PENNSYLVANIA
PHILADELPHIA, PA 19104

BRYAN, CRAIG J., PSYD
ASSOCIATE PROFESSOR
NATIONAL CENTER FOR VETERANS STUDIES
ASSOCIATE PROFESSOR, DEPARTMENT OF PSYCHOLOGY
UNIVERSITY OF UTAH
SALT LAKE CITY, UT 84112

COMTOIS, KATHERINE ANNE JR, PHD, MPH
PROFESSOR
UNIVERSITY OF WASHINGTON
SEATTLE, WA 98195

DAVIS, LORI L., MD
ASSOCIATE CHIEF OF STAFF
TUSCALOOSA VA MEDICAL CENTER
CLINICAL PROFESSOR OF PSYCHIATRY
SCHOOL OF MEDICINE
UNIVERSITY OF ALABAMA
TUSCALOOSA, AL 35404

DOBSCHA, STEVEN K, MD
DIRECTOR, HSRD CENTER TO IMPROVE VETERAN INVOLVEMENT IN CARE (CIVIC)
VA PORTLAND HEALTH CARE SYSTEM
PROFESSOR OF PSYCHIATRY
OREGON HEALTH & SCIENCE UNIVERSITY
PORTLAND, OR 97239

FINLEY, ERIN P, PHD
RESEARCH HEALTH SCIENTIST
SOUTH TEXAS VETERANS HEALTH CARE SYSTEM
ASSOCIATE PROFESSOR
DEPARTMENTS OF MEDICINE AND PSYCHIATRY
UNIVERSITY OF TEXAS HEALTH SCIENCE CENTER
SAN ANTONIO, TX 78229

FORTNEY, JOHN C., PHD
RESEARCH CAREER SCIENTIST
VA PUGET SOUND HEALTH CARE SYSTEM
PROFESSOR
DEPT. OF PSYCHIATRY & BEHAVIORAL SCIENCES
UNIVERSITY OF WASHINGTON
SEATTLE, WA 98195

FRENCH, DUSTIN D., PHD
SENIOR RESEARCH SCIENTIST
EDWARD HINES, JR. VA HOSPITAL
ASSOCIATE PROFESSOR
DEPT. OF OPTHALMOLOGY
NORTHWESTERN UNIVERSITY
CHICAGO, IL 60141

HAAS, GRETCHEN L, PHD
ASSOCIATE DIRECTOR, VISN 4 MIRECC
VA PITTSBURGH HEALTHCARE SYSTEM
ASSOCIATE PROFESSOR OF PSYCHIATRY AND PSYCHOLOGY
WESTERN PSYCHIATRIC HOSPITAL
UNIVERSITY OF PITTSBURGH MEDICAL CENTER
PITTSBURGH, PA 15213

HAMNER, MARK B., MD
STAFF PSYCHIATRIST
RALPH H. JOHNSON VA MEDICAL CENTER
PROFESSOR
DEPARTMENT OF PSYCHIATRY AND BEHAVIORAL SCIENCES
MEDICAL UNIVERSITY OF SOUTH CAROLINA
CHARLESTON, SC 29425

MCINTOSH, SCOTT, PHD
ASSOCIATE PROFESSOR
DEPARTMENT OF PUBLIC HEALTH SCIENCES
UNIVERSITY OF ROCHESTER MEDICAL CENTER
ROCHESTER, NY 14642

PEIRCE, JESSICA M, PHD
ASSOCIATE PROFESSOR
DEPARTMENT OF PSYCHIATRY AND BEHAVIORAL SCIENCES
JOHNS HOPKINS UNIVERSITY SCHOOL OF MEDICINE
BALTIMORE, MD 21224

POLUSNY, MELISSA A., PHD
STAFF PSYCHOLOGIST/CLINICAL INVESTIGATOR
CENTER FOR CHRONIC DISEASE OUTCOMES RESEARCH
MINNEAPOLIS VA HEALTH CARE SYSTEM
ASSOCIATE PROFESSOR, DEPARTMENT OF PSYCHIATRY
UNIVERSITY OF MINNESOTA MEDICAL SCHOOL
MINNEAPOLIS, MN 55417

RAMCHAND, RAJEEV N, PHD
CRAIG NEWMARK FELLOW
BOB WOODRUFF FOUNDATION
NEW YORK, NY 10018

ROLLINS, ANGELA LEIGH, PHD
SUPERVISORY RESEARCH HEALTH SCIENTIST
RICHARD L. ROUDEBUSH VA MEDICAL CENTER
HSRD CENTER FOR HEALTH INFORMATION & COMMUNICATION
ASSOCIATE RESEARCH PROFESSOR
INDIANA UNIVERSITY- PURDUE UNIVERSITY INDIANAPOLIS
INDIANAPOLIS, IN 46202

SAXON, ANDREW J, MD
DIRECTOR, CESATE
VA PUGET SOUND HEALTH CARE SYSTEM
PROFESSOR
UNIVERSITY OF WASHINGTON SCHOOL OF MEDICINE
DEPARTMENT OF PSYCHIATRY AND BEHAVIOR SCIENCES
SEATTLE, WA 98108

SCHUMM, JEREMIAH, PHD
ASSOCIATE PROFESSOR
SCHOOL OF PROFESSIONAL PSYCHOLOGY
WRIGHT STATE UNIVERSITY
DAYTON, OH 45435

WILLIAMS, EMILY CATERINA, MPH, PHD
CORE INVESTIGATOR
VA PUGET SOUND HEALTH CARE SYSTEM HSRD
ASSOCIATE PROFESSOR
DEPARTMENT OF HEALTH SERVICES
UNIVERSITY OF WASHINGTON SCHOOL OF PUBLIC HEALTH
SEATTLE, WA 98108

SCIENTIFIC REVIEW OFFICER

O'BRIEN, ROBERT W., PHD
DEPARTMENT OF VETERANS AFFAIRS
VETERANS HEALTH ADMINISTRATION
OFFICE OF RESEARCH AND DEVELOPMENT
HEALTH SERVICES RESEARCH & DEVELOPMENT
WASHINGTON , DC 20420

Consultants are required to absent themselves from the room during the review of any application if their presence would constitute or appear to constitute a conflict of interest.
